# Liver Grafts for Transplantation from Donors with Diabetes: An Analysis of the Scientific Registry of Transplant Recipients Database

**DOI:** 10.1371/journal.pone.0098104

**Published:** 2014-05-21

**Authors:** Jun Zheng, Jie Xiang, Jie Zhou, Zhiwei Li, Zhenhua Hu, Chung Mau Lo, Weilin Wang

**Affiliations:** 1 Division of Hepatobiliary and Pancreatic Surgery, Department of Surgery, First Affiliated Hospital, School of Medicine, Zhejiang University, Hangzhou, Zhejiang, China; 2 Department of Surgery, The University of Hong Kong, Hong Kong; 3Key Laboratory of Combined Multi-organ Transplantation, Ministry of Public Health Key Laboratory of Organ Transplantation, Hangzhou, Zhejiang, China; University of Colorado School of Medicine, United States of America

## Abstract

Patients with a history of diabetes mellitus (DM) have worse survival than those without DM after liver transplantation. However, the effect of liver grafts from DM donors on the post-transplantation survival of recipients is unclear. Using the Scientific Registry of Transplant Recipients database (2004–2008), 25,413 patients were assessed. Among them, 2,469 recipients received grafts from donors with DM. The demographics and outcome of patients were assessed. Patient survival was assessed using Kaplan–Meier methodology and Cox regression analyses. Recipients from DM donors experienced worse graft survival than recipients from non-DM donors (one-year survival: 81% versus 85%, and five-year survival: 67% versus 74%, *P*<0.001, respectively). Graft survival was significantly lower for recipients from DM donors with DM duration >5 years (*P*<0.001) compared with those with DM duration <5 years. Cox regression analyses showed that DM donors were independently associated with worse graft survival (hazard ratio, 1.11; 95% confidence interval, 1.02–1.19). The effect of DM donors was more pronounced on certain underlying liver diseases of recipients. Increases in the risk of graft loss were noted among recipients from DM donors with hepatitis-C virus (HCV) infection, whereas those without HCV experienced similar outcomes compared with recipients from non-DM donors. These data suggest that recipients from DM donors experience significantly worse patient survival after liver transplantation. However, in patients without HCV infection, using DM donors was not independently associated with worse post-transplantation graft survival. Matching these DM donors to recipients without HCV may be safe.

## Introduction

The growing incidence of type-2 diabetes mellitus (DM) worldwide is recognized as one of the greatest challenges to public health [1]. In 2011, 366 million people worldwide had DM, and this figure is expected to rise to 552 million by 2030 [2]. In general, DM patients suffer worse health-related outcomes than non-DM patients in many medical conditions, and this is also true among liver transplantation (LT) patients [3].

LT is a well-recognized treatment for patients with end-stage liver disease (ESLD) and/or hepatocellular carcinoma (HCC). Ten-year survival of 59% and 83% for deceased donor and living donor transplantation, respectively, has been recorded [4]. In recipients, the role of DM as an independent risk factor for poor survival after LT has been examined explicitly in several studies: a higher mortality in DM patients than in non-DM patients has been observed [3], [5]–[7]. The use of post-transplantation immunosuppressive drugs and other DM-associated factors (e.g., poor wound healing, impaired neutrophil function, obesity, microvascular/macrovascular disease) may result in poor outcomes among DM patients undergoing LT [8]. However, whether or not DM donors have a negative influence on the outcomes of LT recipients is not known. Only a few studies regarding the effect of DM donors on survival have been carried out. Thus, the goal of the present study was to determine if DM donors affected the mortality of LT patients after the procedure.

Based on a national registry database in the USA, we assessed the LT outcomes of patients who received DM donor grafts and compared the results with those who received non-DM donor grafts. We wanted to know whether we could use DM donors safely for patients with ESLD.

## Patients and Methods

### Ethical Statement

The study protocol was approved by the Research Ethical Committee of Zhejiang University (Zhejiang, China). Written informed consent was obtained from all participants. The patient records/information was anonymized and de-identified prior to analysis in the database.

### Data sources

This study used data from the Scientific Registry of Transplant Recipients (SRTR). The SRTR data system includes data on all donor, wait-listed candidates, and transplant recipients in the US, submitted by the members of the Organ Procurement and Transplantation Network (OPTN), and has been described elsewhere. The Health Resources and Services Administration (HRSA), US Department of Health and Human Services provides oversight to the activities of the OPTN and SRTR contractors [9].

### Study cohort

All LT patients who received a first isolated LT between 1 January 2004 and 31 December 2008 were eligible for inclusion into the study. Donors were considered to have DM if positive responses to the variable “donor's history of diabetes” or “donor's duration of diabetes” were recorded. Donors were considered to be non-diabetic if negative responses were recorded for these variables. Recipients for whom a donor history of DM was not known were excluded from the study.

Potentially confounding factors for donors and recipients were examined. Recipient characteristics were: age; sex; ethnicity; history of DM; history of HCC; whether the patients was receiving artificial ventilation; whether the patient was undergoing dialysis in the week before orthotopic liver transplantation (OLT); model for end-stage liver disease (MELD) score; serum levels of creatinine; serum level of bilirubin; and cause of liver disease.

Causes of liver disease were categorized as: hepatitis B virus (HBV); hepatitis C virus (HCV); alcohol; non-alcoholic steatohepatitis (NASH); autoimmune disease (autoimmune hepatitis, primary biliary cirrhosis, primary sclerosing cholangitis); and other causes. Patients classified with HCV in addition to another diagnosis were included under a diagnosis of HCV. Patients who had a diagnosis of HCC were included in the cohort under their primary cause of liver disease.

Donor variables were: age; sex; ethnicity; history of hypertension; body mass index (BMI); donor risk index (DRI) [10]; donation after cardiac death (DCD) donor; warm ischemia time (WIT); cold ischemia time (CIT); and cause of death.

### Outcome measures

The main outcomes were patient survival and graft function. Current status and time-to-outcome were included as outcome measures. Patient follow-up was defined as the time from transplantation until the date of death or last known follow-up. The occurrence and date of death were obtained from data reported by the transplantation centers, and were completed using data from the US Social Security Administration and OPTN.

### Statistical analyses

The study cohort was compared for baseline characteristics with regard to recipients and donors. Statistical analyses were carried out using the Student's *t*-test for continuous variables, and the chi-square test for categorical variables. Survival was assessed using Kaplan–Meier curves and compared with log-rank tests. Cox proportional hazard models were created for the time-to-survival and time-to-graft loss to evaluate the potential predictors of the outcome measures. Variables that were significantly different in the baseline comparison as well as those clinically relevant even if similar at baseline were included in the models. Results were expressed as hazard ratios (HRs) with 95% confidence intervals (CIs). The causes of graft loss and patient death were analyzed and compared between cases and controls. Results are the mean ± standard deviation (SD) unless indicated otherwise. A standard alpha level of 0.05 was taken to indicate statistical significance. All statistical tests were two-sided. Analyses were conducted using SPSS ver15.0 (SPSS, Chicago, IL, USA).

## Results

Out of the 26,645 patients who underwent a first isolated LT during the study period, 25,413 met the inclusion criteria after the exclusion of LTs from donors whose DM histories were not known (n = 1232, 4.6%). Of these, 2469 (9.7%) had DM and 22,944 (90.3%) did not. For the cohorts of recipients with DM donors, the mean duration of follow-up was 32 months. The mean duration of follow-up of recipients with non-DM donors was 34 months.

### Baseline characteristics

The baseline characteristics of recipients from DM donors (n = 2469) and recipients from non-DM donors (n = 22944) are listed in Table 1. DM-donor recipients were older (DM donor 54.0±9.6 years *vs*. non-DM donor 53.0±10.0 years; *P*<0.001) but they (in general) displayed fewer risk characteristics compared with non-DM-donor recipients. They had lower MELD scores (DM donor 20±9 *vs*. non-DM donor 21±9; *P*<0.001), were less likely to be on artificial ventilation (DM donor 3.7% *vs*. non-DM donor 5.0%; *P* = 0.004) and were less likely to be on dialysis 1 week before LT (DM donor 5.8% *vs*. non-DM donor 8.6%; *P*<0.001). There was, however, a greater proportion of HCC recipients in the DM donor cohort (DM donor 25.6% *vs*. non-DM donor 23.6%; *P* = 0.026), whereas the proportion of recipients with DM donors was similar (DM donor 23.8% *vs*. non-DM donor 22.3%; *P* = 0.098). In addition, DM donors were associated with more adverse factors of graft quality, such as donor age (DM donor 54.5±12.9 years *vs*. non-DM donor 40.2±17.2 years; *P*<0.001), history of hypertension (DM donor 78.3% *vs*. non-DM donor 28.2%; *P*<0.001), BMI (DM donor 30±7 years *vs*. non-DM donor 26±6 years; *P*<0.001), DRI (DM donor 2.03±0.42 *vs*. non-DM donor 1.79±0.42; *P*<0.001), and CIT (DM donor 7.6±3.6 h *vs*. non-DM donor 7.4±3.5 h; *P*<0.001). The mean WIT for DM donor transplantation was 42.5±18.8 min, while that of non-DM donors was 41.6±18.9 min (*P* = 0.083). However, DM donor transplantation was associated with a lower prevalence of non-heart-beating donation (NHBD) (DM donor 3.7% *vs*. non-DM donor 5.0%; *P* = 0.003).

**Table 1:** Comparison of patients who underwent transplantation using liver grafts from diabetes mellitus (DM) donors and those who underwent transplantation using liver grafts from non-DM donors with respect to the baseline characteristics of recipients and donors.

Recipient characteristic	DM donors (n = 2469)	Non-DM donors (n = 22944)	P
Age (years)	54.0±9.6	53.0±10.0	<0.001
Male	1725 (69.9)	15,487 (67.5)	0.018
Ethnicity			
White	1799 (72.9)	16,569 (72.2)	0.507
Black	236 (9.6)	2084 (9.1)	0.441
Asian	112 (4.5)	1086 (4.7)	0.686
Hispanic	298 (12.1)	2987 (13.0)	0.186
Other	24 (0.9)	218 (1.0)	0.925
DM	587 (23.8)	5118 (22.3)	0.098
HCC	633 (25.6)	5418 (23.6)	0.026
Cause of liver disease			
HCV	1119 (45.3)	10,459 (45.6)	0.815
HBV	120 (4.9)	1157 (5.0)	0.730
NASH	152 (6.2)	1237 (5.4)	0.111
Alcohol	391 (15.8)	3255 (14.2)	0.027
Autoimmune disease	248 (10.0)	2465 (10.7)	0.304
Other	439 (17.8)	4371 (19.1)	0.131
Serum creatinine (mg/dL)	1.43±1.21	1.54±1.35	<0.001
Serum bilirubin (mg/dL)	7.06±9.78	7.77±10.27	<0.001
On artificial ventilation	92 (3.7)	1154 (5.0)	0.004
Dialysis within 1 week	142 (5.8)	1962 (8.6)	<0.001
MELD score	20±9	21±9	<0.001

Donor characteristic	DM donors (n = 2469)	Non-DM donors (n = 22,944)	P
Age (years)	54.5±12.9	40.2±17.2	<0.001
Male	1375 (55.7)	13,810 (60.2)	<0.001
Ethnicity			
White	1457 (59.0)	15,852 (69.1)	<0.001
Black	558 (22.6)	3433 (15.0)	<0.001
Asian	72 (2.9)	490 (2.1)	0.012
Hispanic	361 (14.6)	2964 (12.9)	0.018
Other	21 (0.8)	205 (0.9)	0.899
BMI (kg/m^2^)	30±7	26±6	<0.001
Hypertension	1933(78.3)	6478(28.2)	<0.001
Cause of death			
Anoxia	523 (21.1)	3338 (14.5)	<0.001
Cerebrovascular accident	1573 (63.7)	9460 (41.2)	<0.001
Head trauma	324(13.1)	9559 (41.7)	<0.001
Other	49 (2.1)	587 (2.6)	0.093
NHBD	91 (3.7)	1156 (5.0)	0.003
DRI	2.03±0.42	1.79±0.42	<0.001
WIT (min)	42.5±18.8	41.6±18.9	0.083
CIT (h)	7.6±3.6	7.4±3.5	<0.001

DM: diabetes mellitus; HCC: hepatocellular carcinoma; HCV: hepatitis C virus; HBV: hepatitis B virus; NASH: non-alcoholic steatohepatitis; BMI: body mass index; NHBD: non-heart-beating donor; DRI: donor risk index; WIT: warm ischemia time; CIT: cold ischemia time.

### Overall graft survival

**Figure 1:**
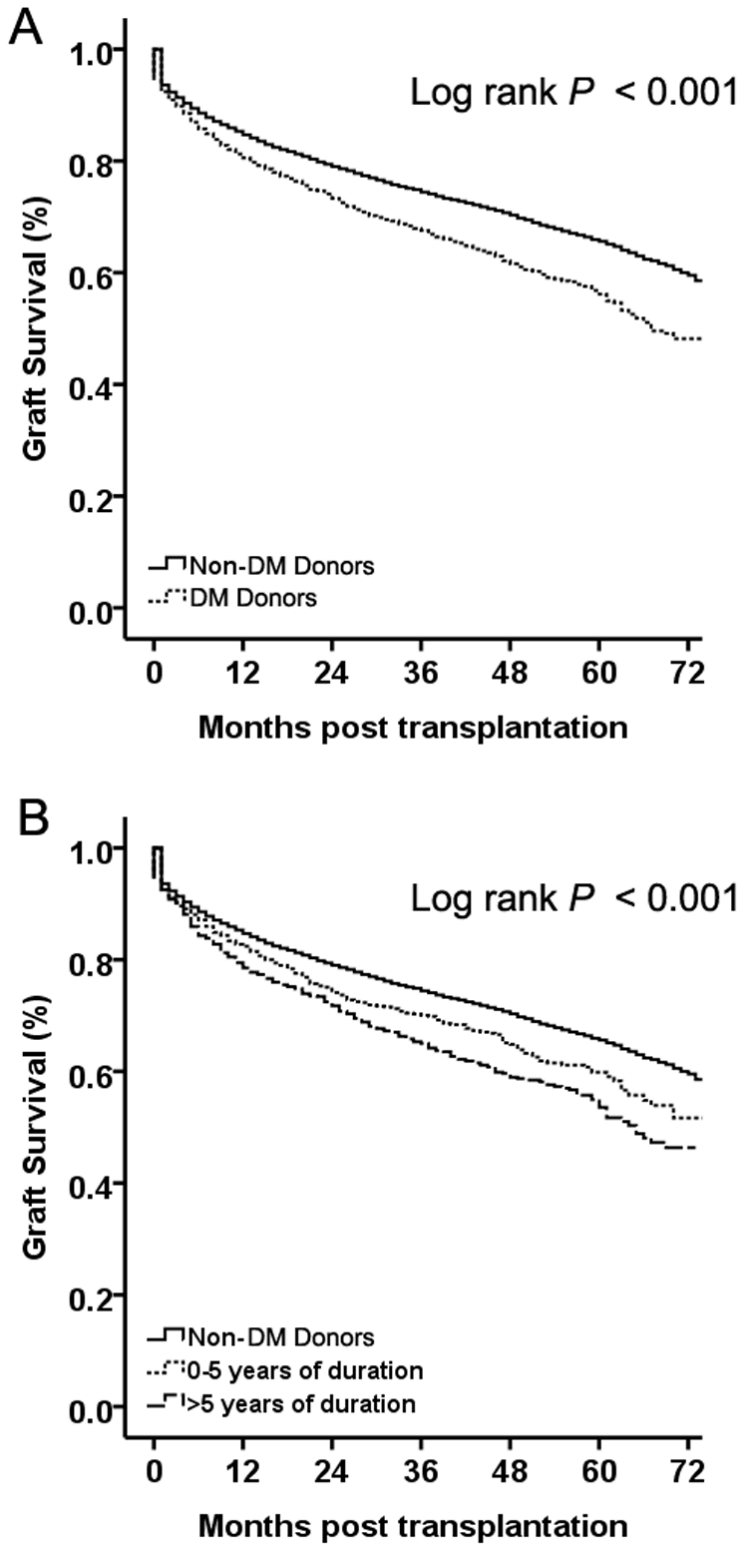
Kaplan–Meier survival curves comparing overall graft survival and graft survival stratified by donor duration of diabetes mellitus (DM) history in recipients from DM and non-DM donors. A) Overall graft survival in recipients from DM and non-DM donors. Recipients with DM donors had significantly lower survival. (B) Graft survival by the duration of DM in the donor. Recipients of liver grafts from DM donors with durations of DM >5 years had significantly lower survival compared with those with durations of DM <5 years and recipients from non-DM donors. Continuous line depicts recipients from DM donors and dashed line depicts recipients from non-DM donors.

A total of 859 (34.8%) recipients from DM donors and 6,382 (27.8%) recipients from non-DM donors lost their grafts. At 1, 5 and 10 years, graft survival was 85%, 74% and 65%, respectively, for recipients from non-DM donors, and 81%, 67% and 56%, respectively, for recipients from DM donors (log rank *P*<0.001) (Fig. 1A).

Data for the duration of DM were available for 2,199 of the 2,469 (89%) donors for whom DM history was known, of whom 1,251 (50.7%) were reported to have durations of DM >5 years. Univariate analyses showed the lowest survival in this category (log rank *P*<0.001) (Fig. 1B).

### Predictors of graft loss at multivariate analyses

Initially, all variables were included in the multivariate analyses, which comprised recipient characteristics (age, sex, ethnicity, cause of liver disease, MELD score, HCC, being on artificial ventilation at the time of LT, dialysis 1 week before LT); and donor characteristics (age, DRI, DCD, history of hypertension).

Table 2 shows the factors identified as significant predictors of graft loss in the entire study cohort using a Cox regression hazard model. DM donors were associated with an increased risk of mortality for patients after LT (HR  = 1.11; 95% CI  = 1.02–1.19). This increased risk of death for recipients from DM donors was comparable in magnitude to other well-known independent risk predictors of graft loss such as MELD at LT (HR  = 1.01 per unit), recipient with HCC (HR  = 1.34), and DRI (HR  = 1.41 per unit). These data showed that a liver graft donated from a subject with DM was an independent risk predictor of graft loss.

**Table 2:** Cox proportional hazard regression analyses to assess predictors of survival of grafts and patients.

Variable	Univariate		Multivariate	
	HR	95% CI	HR	95% CI
DM donors (ref: non-DM donors)	1.24	1.18–1.36	1.11	1.02–1.19
MELD score	1.01	1.01–1.01	1.01	1.01–1.02
HCC (ref: no HCC)	1.15	1.10–1.21	1.34	1.25–1.44
DRI	1.70	1.61–1.79	1.44	1.33–1.57

DM: diabetes mellitus; MELD: Model for End-Stage Liver Disease; HCC: hepatocellular carcinoma; DRI: donor risk index.

### Graft survival as related to allocation of DM donors

**Figure 2:**
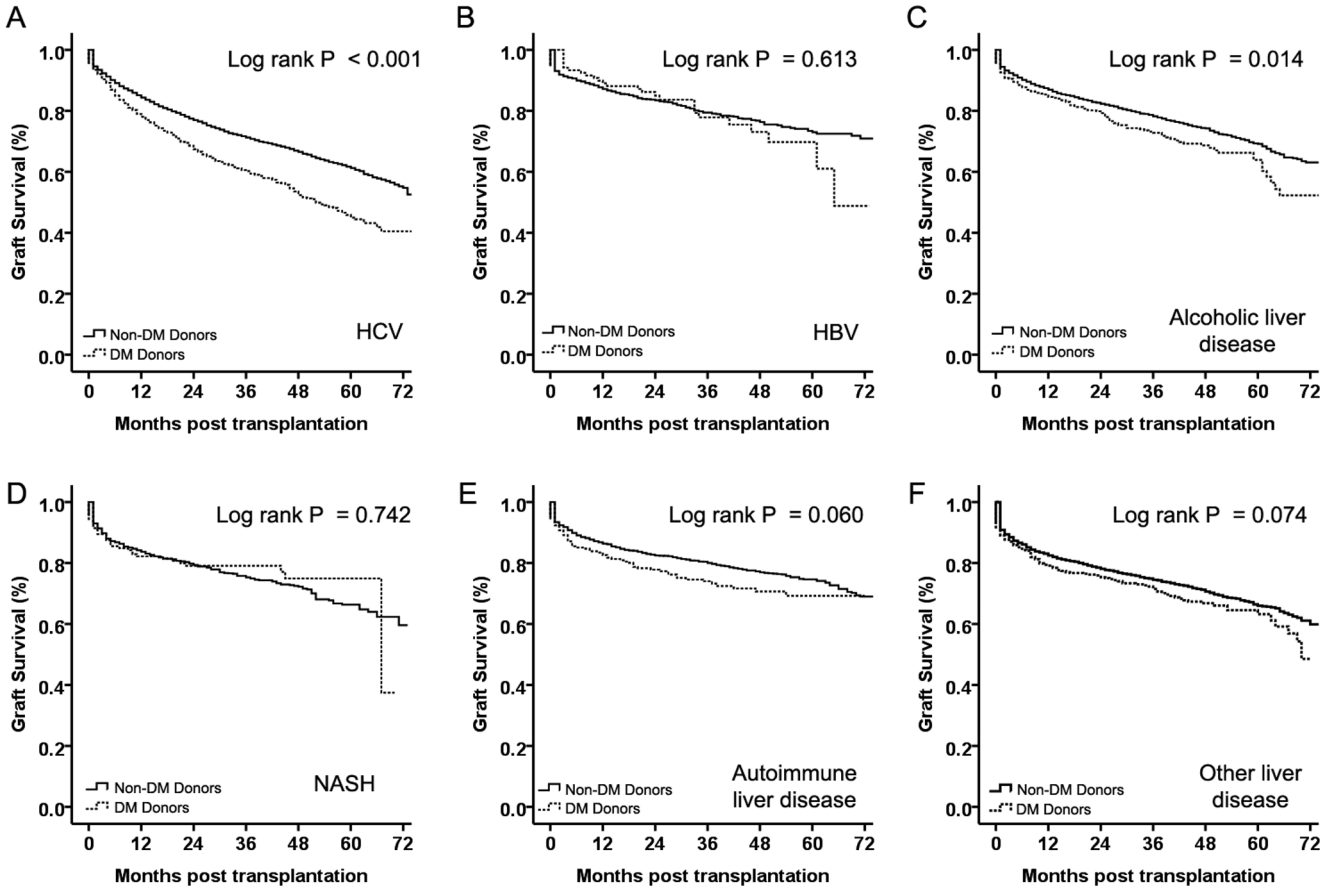
Kaplan–Meier survival curves comparing graft survival of recipients from diabetes mellitus (DM) and non-DM donors according to underlying liver disease. A) HCV, (B) HBV, (C) alcoholic liver disease, (D) NASH, (E) autoimmune liver disease, and (F) other liver disease. Graft survival was significantly lower in recipients from DM donors with HCV infection and alcoholic liver disease. Graft survival was similar between recipients from DM and non-DM donors with HBV infection, NASH, autoimmune liver disease and other liver disease. Continuous line depicts recipients from DM donors and dashed line depicts recipients from non-DM donors.

A liver graft donated from a subject with DM was an independent risk predictor of graft loss, so we explored the effect of DM/non-DM donors on graft survival in relation to the underlying disease of recipients (Table 1). Graft survival from DM donors was worse in recipients with HCV infection, alcoholic liver disease and other liver diseases (Fig. 2A, *P*<0.001; and Fig. 2C, *P* = 0.014; respectively). However, the differences between DM and non-DM donors on graft survival disappeared in recipients with HBV infection, NASH and autoimmune liver disease (Fig. 2B, *P* = 0.613; Fig. 2D, *P* = 0.742; Fig. 2E, *P* = 0.060; and Fig. 2F, *P* = 0.074; respectively).

**Figure 3:**
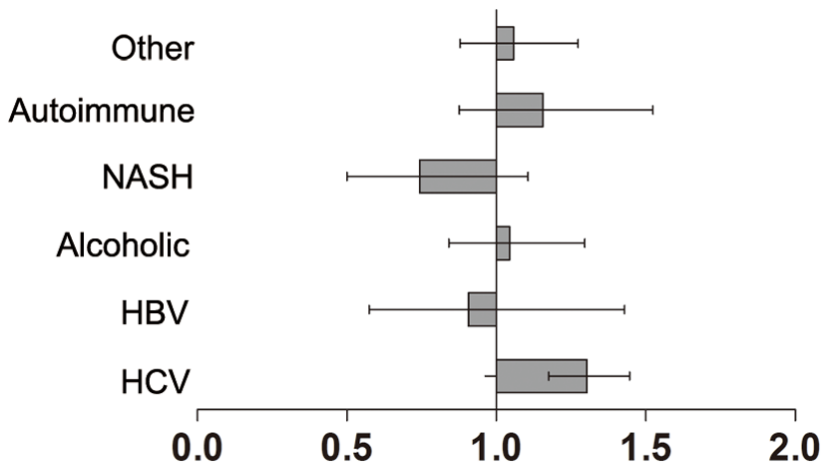
Hazard ratios (with 95% CI) to compare the risk of graft loss after liver transplantation in recipients from diabetes mellitus (DM) donors with various types of underlying liver diseases.

The impact of receiving a liver graft from a subject with DM on graft survival in relation to the underlying disease was investigated further in multivariate analyses. To better understand the effects of DM donors on the various diagnoses studied, we plotted the HR (95% CIs) found in the groups. Only in recipients with HCV was donation from a DM patient an independent predictor of graft loss (Fig. 3).

## Discussion

Using comprehensive clinical data from the SRTR database, the results of the present study suggested that receiving a liver graft from a subject with DM was associated with an increased prevalence of graft loss in a cohort of patients undergoing LT. This increased risk of death remained significant after adjustment for factors for donors and recipients, including those that might represent a selection bias, such as the prevalence of HCC and HCV. Moreover, the present study showed that survival differences were primarily because of lower survival in patients who received liver grafts from donors with a longer duration of DM. If we were to assume that longer duration of DM is a surrogate marker of DM severity, our findings suggest that LT patients who receive liver grafts from subjects with more severe DM may have poorer outcomes. This notion suggests that donors with DM should be employed with caution. This is the first study to compare the outcome of LT recipients from DM donors and non-DM donors.

Even though recipients from DM donors experienced significantly worse graft survival after transplantation compared with those of non-DM donors, a favorable outcome could be expected if a DM donor is transplanted in a favorable condition. Increased risks associated with transplantation for recipients with HCV, HCC or high MELD score have been accepted to extend transplantation to greater numbers of patients in need. We demonstrated that the increased risk of graft loss associated with LT from DM donors was very comparable with the risks associated with these other, well-accepted predictors of risk. Furthermore, by considering the interactions between these risks, a more precise understanding of the impact of receiving a liver graft from a DM donor can be achieved. Increases in the risk of graft loss were noted among recipients from DM donors with HCV at the time of LT, whereas those without HCV experienced greatly improved outcomes (even comparable with recipients from non-DM donors with the same underlying liver disease). In patients without HCV infection before LT, using DM donors was not independently associated with worse graft survival post-transplantation. Therefore, matching DM donors to recipients without HCV may be safe.

Several studies have noted that patients with a history of DM are associated with significant morbidity and mortality post-OLT [3], [6], [11], [12]. However, few studies have addressed the impact on LT outcomes of receiving a liver graft from a donor with DM. Several studies have associated DM with hepatic steatosis, which is a benign form of non-alcoholic fatty liver disease (NAFLD) [13]. Increasing evidence suggests that NAFLD patients with DM are more likely to progress to NASH than NAFLD patients without DM [14], [15]. NASH is a damaging form of NAFLD that leads to fibrosis and cirrhosis, resulting in poor graft function [16]. Studies have shown that grafts with moderate hepatic steatosis (>30%) accelerate the progression of HCV-based disease, and should not be used for HCV patients with high MELD scores [17]. Another study found that moderate steatosis in combination with prolonged ischemic time resulted in worse transplantation outcomes in recipients with HCV [18]. The association of worse graft outcomes in HCV-positive recipients with liver grafts from donors with DM seen in the present study may be attributable to pre-existing graft steatosis and fibrosis induced by DM in the donor, which is further exacerbated by post-transplantation HCV recurrence with subsequent fibrosis.

The number of patients waiting for organ transplantation continues to grow, but donor organs remain in short supply. Over the past few years, steps have been taken to increase the number of organs available for OLT, including using split-LT and introducing living donor LT programs. Marginal donor grafts have also been explored thanks to improvements in surgical methods and immunosuppression. These marginal donors include those with steatotic livers, who are elderly, DCD donors, and donors infected with HCV [19], [20]. Utilization of poorer-quality organs is in response to the increasing divergence between the supply and demand of organs. Improved understanding of the combined risks associated with different factors between donors and recipients is vital to decisions regarding utilization. Transplantation policy should incorporate adequate risk adjustment into measurement of the performance of transplantation centers and into improving informed consent to maximize the individual and societal benefits associated with transplantation.

Due to the registry-based nature of the present study, it had limitations that were mainly related to the data. The most important limitation was the inability to assess steatosis or fibrosis because donor biopsies were not carried out routinely. Secondly, any large database is subject to reporting bias, errors in data entry, and inaccuracies. The SRTR database is not immune to this problem, but these issues may be less of a concern in studies using the SRTR database because of the mandatory participation of all transplantation centers and the electronic editing system, which minimizes data-entry errors. Thirdly, the lack of information on previous and present use of anti-DM drugs as well as liver-biopsy details limited our ability to generalize the results.

The present study also had several extremely important strengths. We included the largest population of patients who have received DM liver grafts with the longest follow-up times available based on the SRTR database (which represents the entire transplantation population in the USA). The large sample size allowed more robust conclusions to be drawn in comparison with previous case reports with smaller sample sizes. Moreover, this is the first survival analysis study concerning post-transplantation graft survival of recipients who received liver grafts from subjects with DM. The SRTR database collects detailed pre-transplantation variables that are known predictors of post-transplantation survival and adjusts for these variables to provide a less confounded assessment of the true effect of receiving a liver graft from a subject with DM on post-transplantation outcome. The limitations mentioned above affected our ability to confirm the reasons underlying our findings, but they did not affect the validity of our primary analyses of the survival of patients and grafts.

In summary, using the largest dataset available for analyses and the longest follow-up periods available to date, we showed that graft survival in recipients with grafts from subjects with DM were worse than those with non-DM grafts when other predictors of post-transplantation survival were taken into account. However, allocating these DM donors to patients without HCV could also result in similar post-transplantation outcomes with non-DM donors. With careful implementation and informed consent from the recipients, matching these DM donors to recipients without HCV may be safe.
