# Correction: Liver Grafts for Transplantation from Donors with Diabetes: An Analysis of the Scientific Registry of Transplant Recipients Database

**DOI:** 10.1371/journal.pone.0106226

**Published:** 2014-08-15

**Authors:** The PLOS ONE Staff

The third affiliation for the authors was omitted. Please refer to the complete affiliations below:

Jun Zheng ^1,2,3^ Jie Xiang ^1,3^ Jie Zhou ^1,3^ Zhiwei Li^1,3^ Zhenhua Hu^1,3^ Chung Mau Lo,^2^* Weilin Wang^1,3*^

^1^Division of Hepatobiliary and Pancreatic Surgery, Department of Surgery, First Affiliated Hospital, School of Medicine, Zhejiang University, Hangzhou, Zhejiang, China

^2^Department of Surgery, The University of Hong Kong, Hong Kong

^3^Key Laboratory of Combined Multi-organ Transplantation, Ministry of Public Health Key Laboratory of Organ Transplantation, Hangzhou, Zhejiang, China.

## References

[1] Zheng J, Xiang J, Zhou J, Li Z, Hu Z, et al (2014) Liver Grafts for Transplantation from Donors with Diabetes: An Analysis of the Scientific Registry of Transplant Recipients Database. PLoS ONE 9(5): e98104 doi:10.1371/journal.pone.0098104 24847864 10.1371/journal.pone.0098104 PMC4029892

